# Lumbago and Its Counterfeits

**Published:** 1902-06-21

**Authors:** 


### Hospital Clinics and Medical Progress.

### LUMBAGO AND ITS COUNTERFEITS.

According to strict definition lumbago is a simple enough affair, and one would not be far wrong in saying that the principal interest in any given case lies, first of all, in the chance that the diagnosis may be wrong; and, secondly, in the question what other mischief is going on, of the existence of which the onset of lumbago is but the more pronounced and obvious indication. If one narrows downs the meaning of lumbago to its dictionary definition, one may take the word as indicating a disorder of the fibro-muscular structures of the loins characterised by local pain and spasm, and a certain degree of fever, and one may add as a qualification that it generally occurs in persons of the "rheumatic diathesis," whatever that term may be regarded as involving. But if, instead of defining what lumbago is, we inquire what may be the meaning of the individual symptoms by which lumbago usually declares itself, we find ourselves in a much wider field; for we have to differentiate the pain from many other forms of pain in that region, the spasm from other causes of spasm, and the fever from the various forms of fever in which pain in the back may be a striking, and for a time, even a predominant feature; while even if we take the fully-formed disease, with all its symptoms neatly and properly displayed, rheumatism is by no means the only condition on which its occurrence may depend, and of which it may be a symptom. A local chill or strain being often the immediate antecedent of an attack of lumbago, the pain, although usually affecting both sides, by no means infrequently falls upon the side which has been more particularly chilled or strained, and the causes of unilateral pain in the lumbar regions are many. First of all one has to consider the possibility of the pain coming from the kidney, and more particularly of its arising from a renal calculus, a thing which may exist for years, giving rise to no symptoms, and then suddenly come into prominence with an attack of pain which may very accurately simulate one-sided lumbago, showing the same relation to bodily movement, the same tendency to spasm, and the same intermission in certain positions of rest. On careful investigation and after several attacks it will usually be found that the locality of the pain is somewhat different, and especially that the direction in which it spreads—when it does spread, which is not always the case at first—will pretty definitely suggest its renal origin; but before the occurrence of a fully developed attack of renal colic there may have been many twinges of "lumbago." Gall-stone, again, is a disease which in certain stages may give rise to intermittent backache. Of course a typical attack of biliary colic clears up the diagnosis at once, but long before this occurs an intermittent "catchy" backache, of the kind commonly but loosely spoken of as "lumbago," may have been present. Even mere flatulence and acidity of stomach may give rise to intermittent backache, which patients often regard as lumbago. It is very difficult to say what symptom may not at one time or another arise in the course of appendicitis, or to exclude a lumbago-like pain in the back as symptomatic of this condition. Then there is a large group of pelvic diseases which, although they do not simulate the more acute and febrile types of lumbago, do undoubtedly give rise to very good imitations of its more chronic and aching, rather than stabbing, forms. Among these in women the first place must be given to cancer of the uterus, while in men the same pre-eminence may be given to that most deceptive condition, cancer of the rectum, a disease which must in all cases be excluded before we can speak positively in regard to an aching and recurring backache for which no definite cause has been discovered. Among women, also, certain displacements of the uterus and its appendages are peculiarly apt to give rise to backache, which may come on quite suddenly and hold them fast very like a "stitch" of lumbago. One need hardly say that lumbar abscess and spinal caries, spinal meningitis, hæmorrhage into the spinal meninges, and other local affections by which the lumbar nerves are irritated or pressed upon may give rise to pains which may have to be differentiated from those of simple lumbago.

Among the more general disorders which may lead to error one has especially to note the tendency of many febrile diseases to give rise to such a degree of backache during the stage of their invasion as to lead a careless observer to put the whole mischief down to "mere lumbago." Just as influenza may begin with intolerable headache and tenderness to light, so may it commence with pain in the back and tenderness to movement, which may well excite a suspicion of lumbago, and the comparatively slight rise in the pulse—as compared with that of the temperature—which sometimes occurs may add to the deception. It is in small-pox, however, that we have the most marked example of the imitation of lumbago by a general febrile disease. For some hours before the disease fully declares itself backache, very like a severe lumbago, may be the predominant symptom. The mistake ought not to occur, but undoubtedly it has occurred again and again.

If now we return to lumbago itself, it is to be noted that, having arrived at the diagnosis that it is lumbago with which we have to deal, we have not arrived at the end of the problem. It is probably true to say that no healthy man suffers from lumbago, and that there is in all cases, as a precedent state, some toxic condition of the blood or tissues, often rheumatic, sometimes gouty, sometimes that form of toxæmia which, from its more prominent symptom, one speaks of as lithæmic, but always a something morbid which tends to manifest itself by inflammation of fibrous tissues when it happens to rise to a certain intensity, and when this happens tends to locate its morbific influence upon some injured spot. An attack of lumbago often appears to be caused by some strain or chill, sitting or standing in a draught, lifting a weight, pulling on one's boots, or what not. But before all this there has usually been present a blood impurity, and, as an immediate precedent, something to give rise to an increase in its intensity, either some over-exertion, bodily or mental, by which more toxin has been produced, or some general chill or hepatic disturbance by which the excretion of the poison has been diminished. Thus it is that lumbago is so often merely symptomatic of some general disturbance of the system; that it is so apt to be brought on by wine, worry, or digestive disorder —just the things that bring on gout; and that it is so apt, when one's blood is in the mood for it, to be set up by slight strains and trivial chills which at other times one would laugh at. Thus also it is that to prevent attacks of lumbago mere coddling is not enough. It is not by insuring constant protection against strains and chills, but by keeping the excretory organs in an active condition that lumbago is best kept at bay. The blood condition has to be got rid of—the recognition of which fact brings much money to many watering places every year.

